# New records of extinct Icacinaceae from Paleocene rainforests of northwestern South America

**DOI:** 10.1002/ajb2.70237

**Published:** 2026-07-16

**Authors:** Laura M. Puente‐Santos, Mónica R. Carvalho, Ethan Shirley, Fabiany Herrera

**Affiliations:** ^1^ Universidad Nacional de Colombia Bogotá Colombia; ^2^ Smithsonian Tropical Research Institute Panama; ^3^ Department of Earth and Environmental Sciences University of Michigan Ann Arbor 48109 Michigan USA; ^4^ Museum of Paleontology University of Michigan Ann Arbor 48109 Michigan USA; ^5^ Earth Sciences, Negaunee Integrative Research Center, Field Museum Chicago 60605 Illinois USA

**Keywords:** Bogotá Formation, Colombia, fossil endocarp, fossil leaves, Icacinaceae, Neotropical rainforest, paleobiogeography, Paleocene, Phytocreneae, X‐ray microcomputed tomography (μCT)

## Abstract

**Premise:**

This study describes three new records of Icacinaceae—*Punctiuga bachue* gen. et sp. nov., *Palaeophytocrene paramoae* sp. nov., and *Goweria bacatana* sp. nov. from the Paleocene (Selandian–Thanetian) Bogotá Formation in Colombia—improving understanding of the distribution and evolution of this family in the Neotropics.

**Methods:**

We analyzed 47 fossil endocarps using microscopy and X‐ray micro‐computed tomography (μCT); we also studied the associated leaf assemblage in search of potential Icacinaceae foliage. We then compared the fossils to extant Icacinaceae specimens from herbaria and the existing literature.

**Results:**

*Punctiuga bachue* features unique morphological traits that resemble and support a close affinity to Iodeae and Phytocreneae. The presence of *Palaeophytocrene paramoae* confirms Phytocreneae diversity in South America during the Paleocene, and the occurrence of leaves of Icacinaceae contribute to the history of Icacinaceae on the continent.

**Conclusions:**

Our results show a previously unacknowledged diversity of Icacinaceae in the Paleocene Neotropics, including two new species of endocarps with affinities to Phytocreneae and Iodeae, clades currently restricted to the Old World tropics. Our findings support an early Paleogene diversification of the family in the Neotropics and the extirpation of tropical lineages in the Neotropics during the later Cenozoic.

Icacinaceae Miers are a family of trees, shrubs, and woody lianas of current pantropical distribution (Kårehed, 2001). Over the past two decades, advances in molecular and morphological phylogenetics have modified the family's classification and clarified their relationships within the Lamiids and Icacinales (Savolainen et al., 2000; Kårehed, 2001; Byng et al., 2014; Byng, 2014; Stull et al., 2015). The traditional concept of Icacinaceae *s.l*., as established by Howard (1940, 1942a, 1942b, 1942c, 1942d, 1943a, 1943b, 1943c, 1992) and Sleumer (1942, 1969, 1971a, 1971b), included 58 genera and ~400 species. This concept changed with the segregation of Metteniusaceae H.Karst. ex Schnizl, Oncothecaceae Kobuski ex Airy Shaw, *Emmotum* Desvaux & Hamilton, Stemonuraceae (M. Roem) Kårehed, and various other genera into Cardiopteridaceae Blume (see Stull et al., 2015). Today, Icacinaceae includes 25 genera and ~200species and, with Oncothecaceae, forms a clade sister to all other lamiids (Angiosperm Phylogeny Group et al., 2016). Four clades are currently recognized within the family, corresponding almost perfectly with the tribal division proposed by Byng et al. (2014): Clade I (Mappieae), Clade II (Icacinae), Clade III (Iodeae), and Clade IV (Phytocreneae) (Angiosperm Phylogeny Group et al., 2016).

The fossil record for Icacinaceae is extensive, dating back to the Late Cretaceous of California (Rozefelds et al., 2021; Del Rio and De Franceschi, 2020a; Atkinson, 2022). The family is well represented in Paleogene deposits, including numerous occurrences of leaves (e.g., Tanai, 1990; Allen et al., 2015), pollen (e.g., Hofmann et al., 2019), flowers (e.g., Del Rio et al., 2017a), wood (Del Rio and De Franceschi, 2020a), and endocarps (e.g., Allen et al., 2015; Stull et al., 2012, 2016; Del Rio et al., 2022). Among these records, fossil endocarps are particularly informative for taxonomic placement and floristic interpretation based on the current understanding of the recognized clades. Their woody nature favors fossilization and their complex morphology allow for identification and comparison across extinct and extant species (Del Rio et al., 2020). As a result, numerous fossil endocarps of Icacinaceae have been identified and described in permineralized and compression floras worldwide, providing a well‐documented history of diversification, particularly during the Paleogene (Figure 1A). Most occurrences come from the Northern Hemisphere (Stull et al., 2011; Stull et al., 2016; Del Rio and De Franceschi, 2020a, 2020b), including from sites with exceptional preservation such as the late Paleocene Rivecourt site (Oise, France; Del Rio et al., 2017b), the early Eocene London Clay Formation (UK; Reid and Chandler, 1933; Chandler, 1961a, 1961b, 1962; Collinson, 1983; Cleal et al., 2001; Stull et al., 2016), the middle Eocene Clarno Nut Beds (Oregon, USA; Manchester, 1994), the middle Eocene Messel biota (Germany; Collinson et al., 2012), and other Eocene deposits across Europe (Kvaček and Bůžek, 1995; Del Rio and Franceschi, 2020b). This extensive record shows that the number of species and morphological variation of Icacinaceae increased during the Paleocene and peaked during the Eocene, particularly in Boreotropical forests (Pigg et al., 2008; Stull et al., 2011; Del Rio, 2018). Boreotropical forests were extensive across North America, Europe, and Asia, enabling species exchange, especially around the Paleocene‐Eocene climatic optima (Del Rio and De Franceschi, 2020a). Fossil occurrences from this time, such as those from Europe and the Americas, reveal a rich representation of genera of Icacinaceae. The distribution and diversity of Icacinaceae contracted during the Oligocene, likely owing to global cooling trends and the consequent decline of Boreotropical forests (Del Rio and De Franceschi, 2020a).

**Figure 1:**
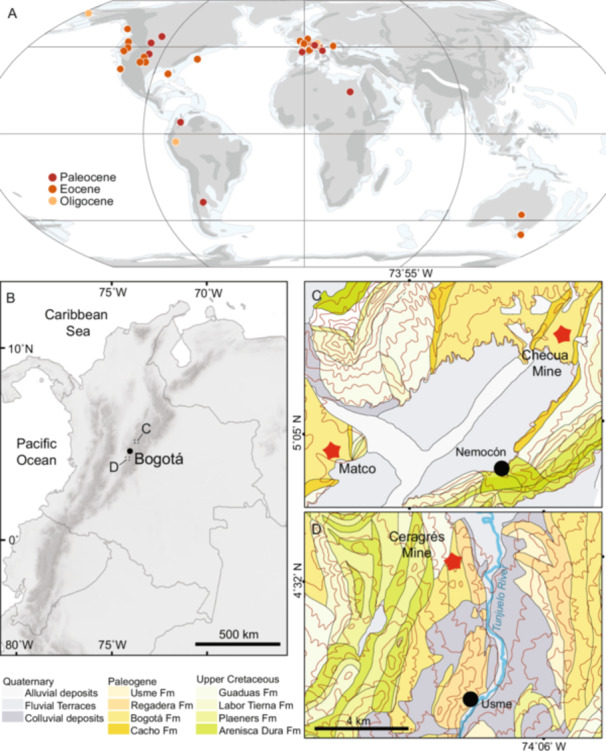
Reported occurrences of endocarps and flowers of Icacinaceae in Paleogene deposits. (A) Paleogene paleogeography depicting occurrences of Icacinaceae. Data points are based on Del Rio and De Franceschi (2020a), Rozefelds et al. (2021), and Poore et al. (2023). Paleogeography is based on reconstructions of the early Eocene from Cao et al. (2017). (B) Collection sites for *Punctiuga bachue* gen. et sp. nov., *Palaeophytocrene* sp., and *Goweria bacatana* sp. nov. in Cundinamarca, Colombia. (C) Simplified geological map of Nemocón and surrounding areas depicting sites for fossiliferous localities in Checua (MC2310) and Matco siltstone mines. (D) Geological map of Usme and Mochuelo localities, depicting Ceragrés brickworks mine (fossil locality MC1813). Geological maps modified from Gómez Tapias et al. (2023).

Despite the modern pantropical distribution of Icacinaceae, their fossil record in low and southern latitudes remains sparse. Paleogene records of Phytocrenae from Australia (Rozenfelds et al., 2021) and Patagonia (Berry, 1937; Poore et al., 2023) suggest high‐latitude connections across the southern continents and imply that Icacinaceae were likely present on all seven continents in the Cenozoic—including Antarctica, although endocarps have yet to be found there. The biogeographic affinities of low‐latitude taxa, however, remain poorly understood. In tropical South America, fossils of Icacinaceae include at least four and likely more species in Phytocrenae: *Palaeophytocrene hammenii* Stull and two unidentified species in *Palaeophytocrene* Reid & Chandler and *Phytocrene* Wall from the Paleocene Bogotá and Cerrejón Formations in Colombia; *Pyrenacantha austroamericana* from the Oligocene Belén Flora in Perú (Stull et al., 2012); and a number of undescribed icacinaceous endocarps from the Early Miocene of Panama (Herrera, 2014). These sparse but widespread occurrences highlight the lack of information on the historical distribution of this family in the region.

Here, we present the description of a new species in Icacinaceae with affinities to the clade formed by Iodeae+Phytocrene, *Punctiuga bachue* gen. et sp. nov., based on fossil fruit compressions from the Paleocene Bogotá Formation in Colombia. In addition, we describe *Palaeophytocrene paramoae* sp. nov. (Phytocreneae) and leaves of *Goweria bacatana* sp. nov. with affinities to Icacinaceae from the same depositional unit. These new findings highlight a previously unrecognized diversity of Icacinaceae in tropical America during the Paleocene and contribute to the history of the family in low‐latitude forests.

## MATERIALS AND METHODS

### Geological and paleobiological setting and specimens

The Bogotá Formation is a 1.5‐km‐thick sedimentary sequence of claystones, mottled mudstones and siltstones, interlayered sandstones, and occasional conglomerates and breccias (Morón et al., 2013). These sediments were deposited during the Paleocene (Selandian–Thanetian) and Eocene (Ypresian) in fluvial and lowland environments, preceding the Andean uplift in northwest South America (Bayona et al., 2008, 2010). This sedimentary unit is now exposed along the Eastern Cordillera in central Colombia. It contains a diverse array of vertebrate fossils, including pleurodire turtles (Cadena, 2014), mammals (Villarroel, 1987), crocodylians, boid snakes, frogs, lizards, and dipnoan fishes (Bloch et al., 2008). It also features a diverse assemblage of leaf, flower, fruit, and seed compressions (Herrera, 2014; Carvalho et al., 2021a; Herrera et al., 2011, 2019, 2024; Puente‐Santos et al., 2026). The leaf flora of the Bogotá Formation has been interpreted as an early example of a Neotropical rainforest, based on the abundance of macrophylls and megaphylls with drip tips, mean annual precipitation estimates of >180 cm, and family‐level composition resembling that seen in living rainforests (Carvalho et al., 2021b). The strata containing plant fossils are dated as Paleocene (Selandian–Thanetian) on the basis of palynological assemblages and biostratigraphic correlation (Carvalho et al., 2021b).

### Specimen imaging and description

We analyzed a total of 47 endocarps and 23 leaf compressions. The specimens were collected from the Checua (Nemocón, Colombia; 5.135725°, −73.846711°), Matco (Cogua, Colombia; 5.076700°, −73.955300°), and Ceragrés mines (Cogua, Colombia; 4.534366°, −74.139274°), which exploit claystone and siltstone deposits of the Bogotá Formation in central Colombia (Figure 1B–D). Endocarp specimens are housed at the Universidad del Rosario (Bogotá, Colombia) under the collection numbers UR‐CP‐0621 to UR‐CP‐0667, while leaf fossils are housed at Universidad de Caldas in Manizales, Colombia (STRI 45845, 47091, 47096–47102) and Universidad Eafit in Medellín, Colombia (STRI 12462–12473, 12673, 12674, 13023).

We photographed each fossil with a Canon R6 camera, using a controlled setup to optimize lighting and focus. Given the three‐dimensional nature of the endocarp compressions, multiple images were taken at different focal planes with the aid of Helicon Remote version 4.5 (Helicon Soft, Kharkiv, Ukraine) and Z‐stack using Helicon Focus version 8.2.17 (Helicon Soft). Photographs were edited to enhance brightness and contrast and improve the visual presentation of key anatomical features using Adobe Photoshop version 25.12 (Adobe, San Jose, California, USA). We followed the *Manual of Leaf Architecture* (Ellis et al., 2009) for general leaf traits and Del Rio (2018) for descriptions of features of endocarp morphology.

### Endocarp comparative morphology

We compared our fossil endocarps with published descriptions (Del Rio et al., 2020) and herbarium specimens of Icacinaceae housed at the Colombian National Herbarium (COL) and the Herbarium of the University of Michigan (MICH). Three well‐preserved fossil endocarp specimens (UR‐CP‐0626, UR‐CP‐0632, UR‐CP‐0634) and four representative endocarps of extant Icacinaceae (MICH1461090, MICH1506112, MICH1506119, MICH1461144) were scanned using X‐ray micro‐computed tomography (Nikon XT H 225ST μCT scanner) at the CTEES facility of the Department of Earth and Environmental Sciences, University of Michigan, Ann Arbor, Michigan, USA. Endocarps were scanned at 177 kV and 177 μA at 4 s exposure timing, using a copper filter of 1 mm. Resolution of voxel x = y = z = 49 µm with 2000 projection images was achieved. This technique allowed for a nondestructive analysis of the endocarps’ internal anatomy and was particularly useful for identifying key diagnostic traits, such as the presence and arrangement of vascular bundles, the thickness of the endocarp wall, and the nature of the internal cavity. The high‐resolution scans provided three‐dimensional data, which were further processed to enhance visualization. To refine the interpretation of internal structures, µCT scans were manually segmented using Amira version 2019.1 (Thermo Fisher Scientific, Waltham, Massachusetts, USA).

Endocarps were described following the criteria established by Del Rio et al. (2020), ensuring a standardized approach for Icacinaceae. This framework provided a comparative basis for distinguishing the fossil endocarps from extant and other fossil representatives of the family. Measurements of key morphological features, including endocarp length, width, wall thickness, and surface ornamentation, were obtained and quantified using ImageJ (Schneider et al., 2012), ensuring precision and consistency in data collection while minimizing observational biases.

## SYSTEMATICS AND RESULTS

***Family***—Icacinaceae Miers.

***Genus***—*Punctiuga* Puente‐Santos, L.M & M. Carvalho gen. nov.

***Generic diagnosis***—Endocarps elliptical to globose in lateral view, with an asymmetrical apex and a rounded, symmetrical base. Keel present along the endocarp plane of symmetry from apex to base. Vascular bundle embedded within the endocarp wall. Outer endocarp surface ridged and pitted, with numerous longitudinal ridges extending along its length. Vasculature resting on the ridges and going through some pits.

***Etymology***—The genus name *Punctiuga* is derived from the Latin *puncta* (“dots”) and *iuga* (“ridges”), referring to the presence of two distinctive characters in its outer surface, ridges, and pits.

***Species***—*Punctiuga bachue* Puente‐Santos, L.M & M. Carvalho sp. nov.

***Specific diagnosis***—Endocarps elliptical. Outer surface with five or six main longitudinal ridges and with pits. Areoles are present and formed by the intersection of ridgelets that interconnect with the main ridges. Keel surrounding the endocarps in the plane of symmetry. Vascular bundle embedded in the endocarp wall and running from the base to the apex along the plane of symmetry. Locule surface papillate.

**Figure 2:**
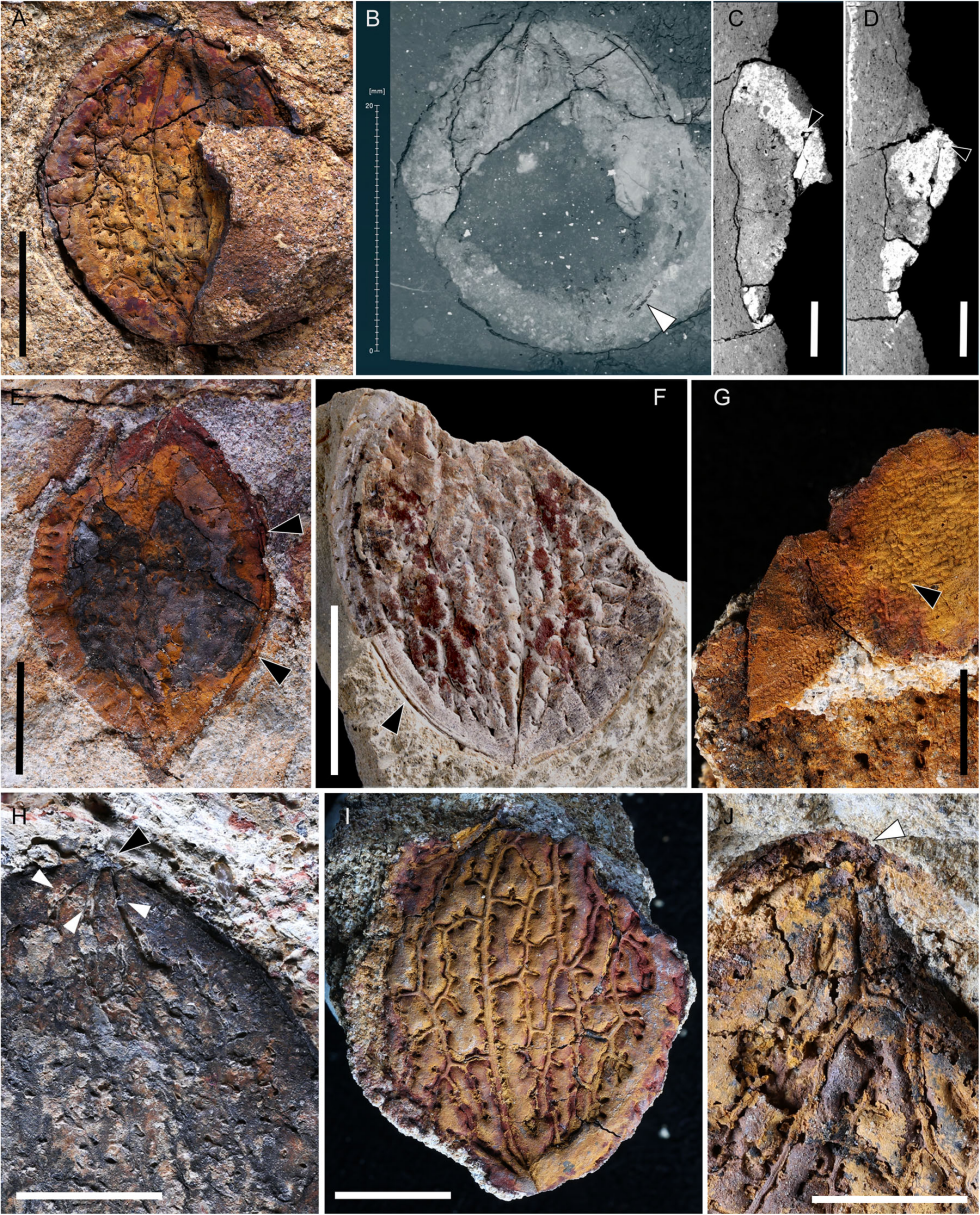
*Punctiuga bachue* Puente‐Santos, L.M. & M. Carvalho gen. et sp. nov. morphology. (A) Holotype UR‐CP‐0626. (B) Volume rendering of µ‐CT scanning of UR‐CP‐0626 showing interior of the fossil compression. White arrow shows the vascular bundle that runs along the ventral side of the endocarp. (C, D) Optical cross‐sections of UR‐CP‐0626. Arrows show a vascular bundle embedded within the keel. (E, F) Rounded (specimen UR‐CP‐0659, F) to slightly asymmetric (specimen UR‐CP‐0650, G) endocarp shape. Arrows highlight continuous vascular bundle running along the ventral side of the endocarp. (G) Specimen UR‐CP‐0650. Endocarp infilled with sediment, showing papillae impressions (arrow). (H) Specimen UR‐CP‐0631. Black arrow indicates the entry point for the vascular bundle into the endocarp; white arrows show three main veins that branch off upon entry into the endocarp wall. (I) Specimen UR‐CP‐0640 showing organized, regular reticulation of vasculature. (J) Specimen UR‐CP‐0634. Arrow indicates the entry point for the vascular bundle into the endocarp. Scale bars = 1 cm (A, E, F); 3 mm (G); 5 mm (C, D, H, I, J).

***Description***—*Punctiuga bachue* is described on the basis of 38 endocarp compressions. The endocarps are elliptical to globose in lateral view and unilocular (Figure 2A,E,G). They range in length from 28 mm to 35 mm and in width from 13 mm to 24 mm (Figure 2E,G). All endocarps show a conspicuous endocarp wall 3.0–3.6 mm thick. The base is rounded to acute in outline, while the apex appears slightly asymmetrical (Figure 2J). The outer surface is characterized by a complex ornamentation of ridges and pits (Figure 2A,F,I). Five or six longitudinal ridges run along the length of the endocarp and extend nearly the full length of the endocarp, with a main one that reaches the base. Smaller ridgelets interconnect principal ridges, forming polygonal, elongated areoles (Figure 2I). Interconnecting ridgelets also form areoles of irregular size and shape that enclose free terminations (Figure 2I). Remnants of the endocarp's surface vasculature are visible along the ridges and extend into some of the pits, creating a mesh‐like reticulum (Figure 2H,I). Pits protrude only halfway into the endocarp wall (Figure 2E). The locule surface is finely papillate (Figure 2G). A keel encircles the endocarp along its plane of symmetry (Figure 2F). The vascular or funicular bundle is ~0.62 mm thick, embedded within the endocarp wall (Figure 2C,D), and extends internally from the base to the apex (Figure 2B,E,F), where it ultimately enters the endocarp apically, lacking evidence of a distinct pore (Figure 2J).

***Etymology***—The epithet *bachue* honors the Muisca (Pre‐Columbian culture) goddess Bachué, mother of humankind and germination in nature. According to mythology, she taught the first inhabitant children of the Bogotá savanna how to cultivate the land. The Muisca were a highly prosperous indigenous civilization that inhabited the highlands of Cundinamarca and Boyacá, and southern Santander in Colombia, from the sixth century BCE to the present day.

***Holotype***—UR‐CP‐0626 (Figure 2A).

***Paratypes***—UR‐CP‐0625, UR‐CP‐0632, UR‐CP‐0639, UR‐CP‐0640, UR‐CP‐0643, UR‐CP‐0650.

***Type locality and age***—Locality MC2310 (5.135725°, −73.846711°), Checua mine, Bogotá Formation, Paleocene (Selandian–Thanetian), Nemocón, Cundinamarca, Colombia.

***Repository***—Museo de Historia Natural, Universidad del Rosario, Bogotá.

***Systematic affinity***—The fossil endocarps examined exhibit a combination of traits that strongly support their classification within the family Icacinaceae. Members of this family share fruits with bilaterally symmetrical, unilocular, and single‐seeded endocarps that are elliptical to globose in lateral view and lenticular to globose in transverse section (e.g., Del Rio et al., 2020). A keel is often present, surrounding the endocarp in the plane of symmetry, though it can sometimes be faint or accompanied by a channel. The apex varies from acute to flattened and is often asymmetrical or slightly asymmetrical, while the base is usually symmetrical or cleft on one side, ranging from rounded to acute. The outer surface of Icacinaceae endocarps can be smooth or, more commonly, rugose, ridged, or pitted, frequently exhibiting a combination of ridges and pits (Del Rio et al., 2020).

Despite the overall distinctiveness of Icacinaceae endocarps, most endocarp traits, such as the type of surface ornamentation and vascular patterns, are highly convergent across the family and rarely diagnostic in isolation. Smooth and rugose surfaces occur across all four tribes of Icacinaceae (Del Rio et al., 2020), which suggests that these traits may represent plesiomorphic conditions. Ridged morphologies, such as those characterizing *Punctiuga bachue*, are observed among members of Iodeae, Icacineae, and Phytocreneae but are most prominent among Iodeae and Icacineae, indicating a possible synapomorphy that unites these three groups. Endocarps with an outer surface that bears pits exclusively are restricted to Phytocreneae (excluding *Sarcostigma* Wight & Arn; Sleumer, 1971; Stull et al., 2012; Del Rio et al., 2020; Del Rio and De Franceschi, 2020a) and may represent a synapomorphy within the group.

**Figure 3:**
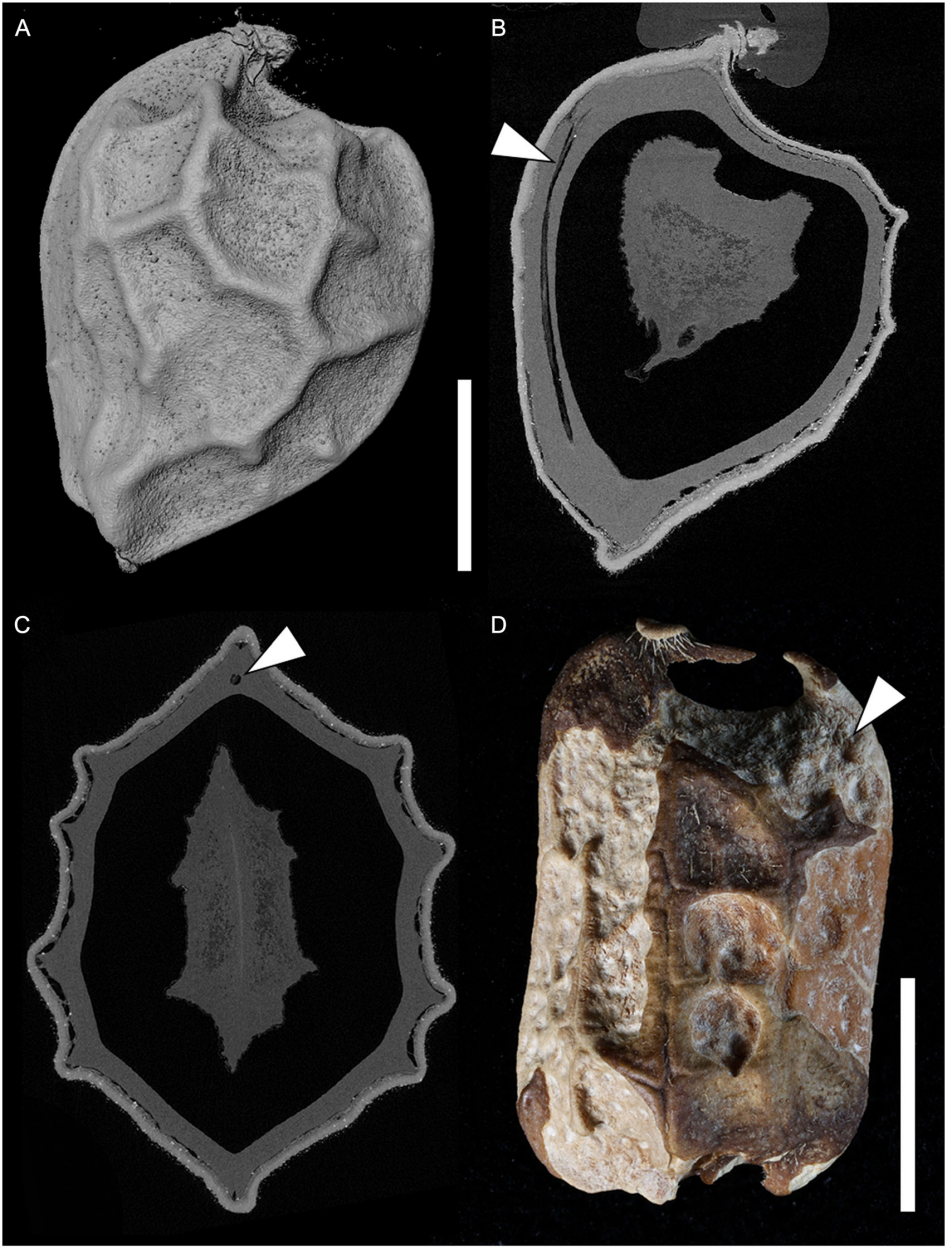
Endocarp morphology of extant *Iodes*. (A) Isosurface reconstruction of *Iodes ovalis* (MICH1461090) showing external ridged morphology. (B) Virtual cross section of *I. ovalis*. Arrow indicates vascular bundle running along from the base to apex, along the plane of symmetry. (C) Virtual transversal section of *I. ovalis*. Arrow indicates vascular bundle embedded in the endocarp wall. (D) External morphology of *I. phillipinensis* (MICH1461095) showing external ridges and pits (arrow). Scale = 5 mm.

Together, endocarp surface morphology and vascular patterns aid in formulating hypotheses of natural affinities. The combination of a ridged ornamentation (Figure 3), a vascular bundle that is embedded in the endocarp wall (Figure 3), and papillae in the inner locule is unique to *Iodes* (Figure 3; Stull et al., 2011, 2016; Del Rio et al., 2019) and has been used as diagnostic in recent revisions of fossil Icacinaceae (Allen et al., 2015; Del Rio and De Franceschi, 2020a). This combination of traits is also present in the endocarps of *Punctiuga bachue* and is suggestive of an affinity to *Iodes*. Nonetheless, the South American fossils differ from *Iodes* in their large size and thick endocarp wall and in having deep pits in addition to ridges on their outer surface (Figure 2E,G). The combination of large size and relatively thick endocarp wall is unusual among extant and fossil *Iodes*. Furthermore, while a few extant *Iodes* species have pits on their outer surface in addition to their ridged ornamentation (Figure 3), these pits are distinctively shallow and do not protrude into the endocarp wall, as observed in *P. bachue* (Figure 2E,G). Deeply protruding pits are not common among Icacinaceae and are observed strictly in endocarps of members of Phytocreneae (Sleumer, 1971; Stull et al., 2012; Del Rio et al., 2020; Del Rio and De Franceschi, 2020a).

*Punctiuga bachue* shows a combination of traits that may be interpreted as “intermediate” between derived genera of Phytocreneae and members of Iodeae (i.e., *Iodes*). Given that these two groups have been consistently recovered as sister clades in recent phylogenetic analyses (Stull et al., 2015), we interpret this taxon as a stem group member of Phytocreneae+Iodeae.

**Comparison to fossil Icacinaceae**—Fossil endocarps of Icacinaceae can be generally divided into two major groups: those with pitted ornamentation (“Phytocreneae‐like”) and those with a reticulate or ridged ornamentation. Among the fossil taxa that share a ridged ornamentation, endocarps of *Iodes* and *Iodicarpa* Manchester are unique within Icacinaceae in also having a vascular bundle embedded within the endocarp wall and a papillate locule lining (Del Rio and Franceschi, 2020), although some extant species lack the latter (Allen et al., 2015). *Iodicarpa* was circumscribed to include fossil endocarps that resembled *Iodes* in “general form, endocarp wall composed of digitate cells, and papillate inner locule lining” (Manchester, 1994) but differed in their larger size and thicker endocarp wall. The type species, *Iodicarpa ampla* Manchester, has the ridged outer surface, vascular (funicular) bundle embedded in the endocarp wall, papillate locular lining that characterize *Iodes*, supporting the recognition of *Iodicarpa* as a fossil genus of endocarps similar to but differing from *Iodes* (Manchester, 1994).

Among fossil Icacinaceae, *Punctiuga bachue* is most similar to fossil *Iodes* and *Iodicarpa* in sharing a ridged ornamentation, papillate inner locule, and vascular bundle inside the endocarp wall. Nonetheless, the occurrence of deep pits in the endocarp wall of *P. bachue* is a clear distinction between these taxa, as both *Iodes* and *Iodicarpa* lack this feature (Del Rio and Franceschi, 2020). In fact, *P. bachue* is the first to have both a ridged morphology and a deeply pitted surface, in combination with a vascular bundle embedded in the endocarp wall and a papillate inner locule. This specific combination of traits supports the description of *P. bachue* as a new genus and species.

***Tribe***—Phytocreneae Engler.

***Genus***—*Palaeophytocrene* Reid & Chandler.

***Species***—*Palaeophytocrene paramoae* Puente‐Santos, L.M & M. Carvalho sp. nov.

***Specific diagnosis***—Endocarp elliptical; outer surface covered in ~60 deep, polygonal to rounded, pits of variable size, irregularly spaced and associated with shallow mounds; apex shape symmetrical, moderately elongated; base rounded.

***Description***—This taxon is described on the basis of a single endocarp (Figure 3) that is 32 mm long and 17 mm wide. Its outer surface is deeply pitted, with ~60 pits covering each face. The apex is asymmetrical, while the base appears rounded. The pits seem to be associated with shallow mounds and exhibit a polygonal to rounded shape. Some of these pits are longer than they are wide and extend into the locule cavity. The tubercles range in length from 2.9 mm to 6.9 mm and in width from 1.4 mm to 2.2 mm. No visible pores are present.

***Etymology***—This species is named in honor of the late María Páramo, Colombian paleontologist who dedicated her life to the study of Cretaceous marine reptiles in Colombia.

**Figure 4:**
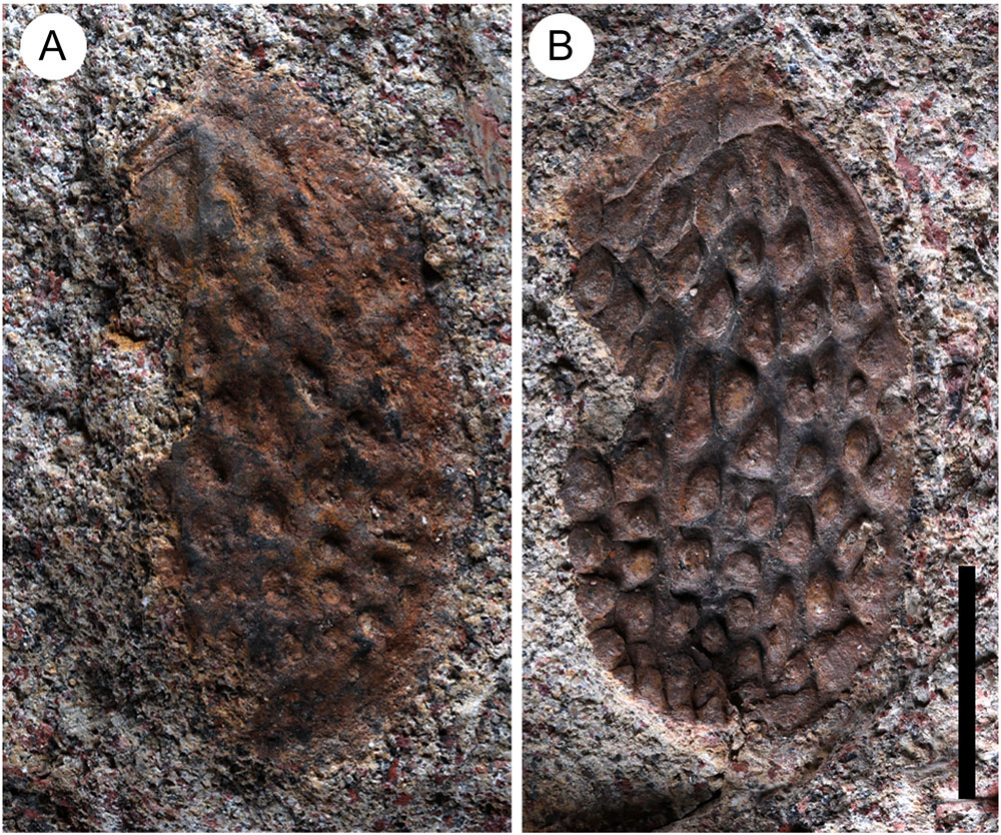
Holotype of *Palaeophytocrene paramoae* (UR‐CP‐0630). (A) Part and (B) counterpart of UR‐CP‐0586. Scale = 1 cm.

***Holotype***—UR‐CP‐0630 (Figure 4).

***Type locality and age***—Locality MC2310 (5.135725°, −73.846711°), Checua mine, Bogotá Formation, Paleocene (Selandian–Thanetian), Nemocón, Cundinamarca, Colombia.

***Repository***—Museo de Historia Natural, Universidad del Rosario, Bogotá.

***Systematic affinity***—The fossil specimen described here is assigned to *Palaeophytocrene*, based on the presence of protruding mounds, a characteristic diagnostic of the genus. *Palaeophytocrene paramoae* is represented by a single, nearly complete endocarp compression that differs from *P. hammenii*, an endocarp species previously described from the Bogotá Formation (Stull et al., 2012). This new specimen (Figure 4) is distinct from *P. hammenii* (Stull et al., 2012) in its larger size (32 mm long and 17 mm wide vs. ~5 mm long and 3.25 mm wide), its elliptical rather than rounded shape, and the presence of abundant, irregularly distributed tubercles. The combination of these traits supports its placement within *Palaeophytocrene*, while also suggesting that it represents a species distinct from *P. hammenii*.

Within Icacinaceae, Phytocreneae forms a clade that is well supported by both molecular and morphological features (Stull et al., 2015; Del Rio et al., 2020). With the exception of *Sarcostigma*, the endocarps of Phytocreneae are characterized by a distinctive pitted outer surface and tubercles that protrude into the locule to varying extents (Sleumer, 1971; Stull et al., 2012; Del Rio et al., 2020; Del Rio and De Franceschi, 2020a). Additionally, the endocarps of Phytocreneae exhibit a pair of symmetrical pores, positioned eccentrically and subapically on each of the endocarp faces, while their vascular bundle is located outside the endocarp wall (Del Rio et al., 2020).

In the fossil record, the genus *Palaeophytocrene* (Reid and Chandler, 1933; Stull et al., 2016) is distinguished from its extant counterpart by the extent to which tubercles protrude into the locule. Whereas endocarps of *Palaeophytocrene* have tubercles that protrude beyond the endocarp wall and extend into the locule, tubercles do not protrude beyond the endocarp wall in endocarps of living *Phytocrene* and are instead described as shallow mounds (Del Rio et al., 2020).

***Genus***—*Goweria* Wolfe.

***Species***—*Goweria bacatana* Puente‐Santos, L.M & M. Carvalho sp. nov.

***Specific diagnosis***—Leaves notophylls to mesophylls, petiolate. Lamina elliptical, unlobed and untoothed, symmetrical to medially asymmetrical, with length‐to‐width ratios of 2:1. Leaf apex acuminate, base rounded, basal insertion symmetrical to asymmetrical. Venation pinnate, secondary veins brochidodromous, crowded toward the base. Agrophics simple. Tertiary veins are percurrent, proximally chevroned, and distally straight. Quaternary veins alternate percurrent.

**Figure 5:**
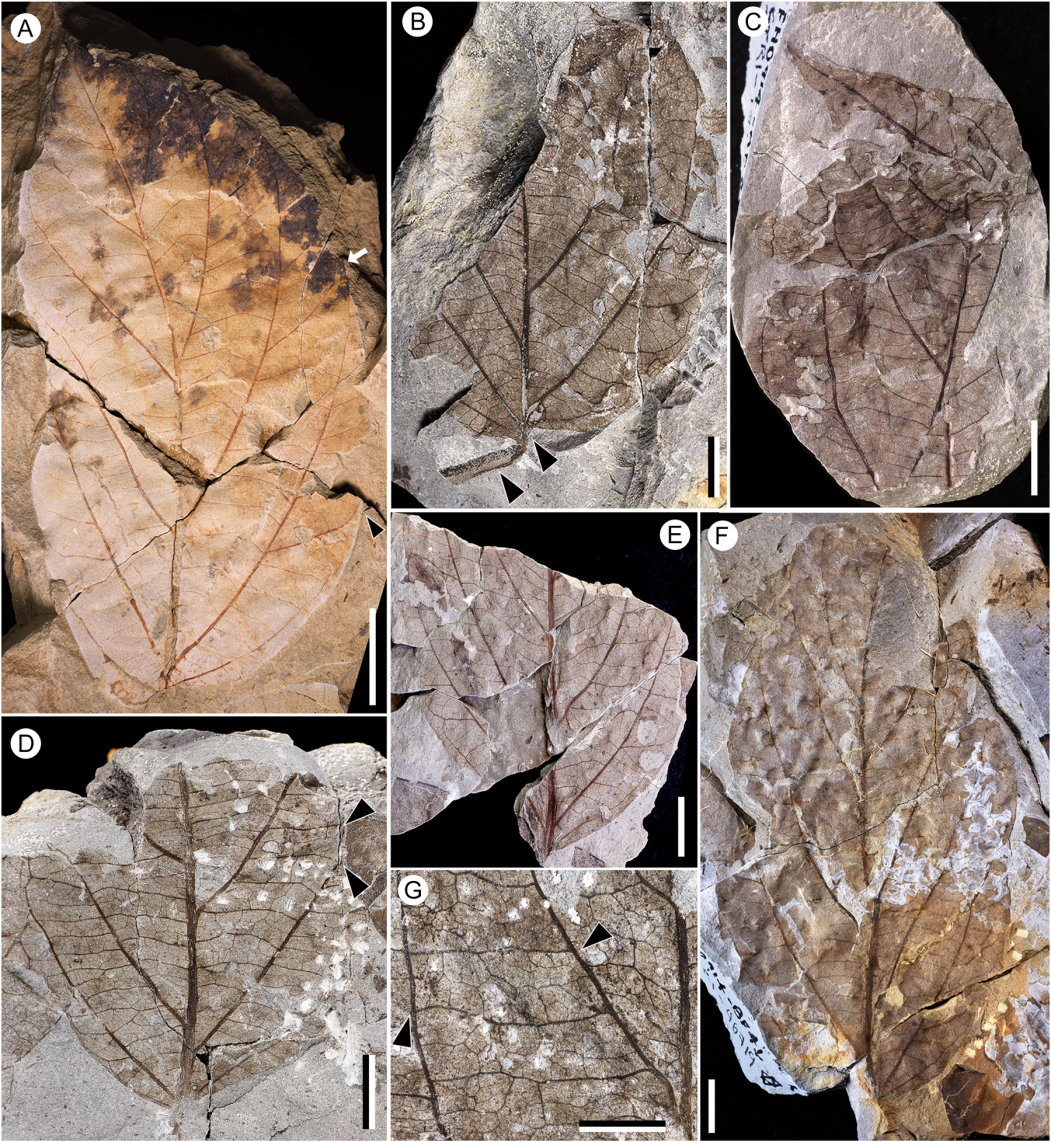
*Goweria bacatana* Puente‐Santos, L.M. & M. Carvalho sp. nov. morphology. (A) Holotype (STRI 45854) showing a curved, slightly asymmetric leaf lamina. White arrow indicates compound agrophic veins. (B) Detail of petiole and base shape (STRI 12472). Arrow highlights basal asymmetric insertion and apparent petiolar vascular strand. (C) Leaf fragment showing detail of an acuminate apex (STRI 12470). (D) Leaf base (STRI 12473). Arrows show tertiary veins varying from chevroned to straight percurrent. (E) Leaf fragment (STRI 12467) showing a nearly symmetric leaf base. (F) Example of nearly symmetric leaf lamina (STRI 46719). (G) Detail of percurrent tertiary and alternate percurrent quaternary venation (STRI 12468). Scale bars = 2 cm (A); 1 cm (B, C, D, E, F); 5 mm (G).

***Description***—*Goweria bacatana* (Figure 5) is described on the basis of 23 complete or partially complete leaf impressions. The leaves are notophylls and mesophylls, with laminas 10.5–16.3 cm long and 5.3–8.6 cm wide, untoothed, elliptical in shape, with an acuminate apex (Figure 5C). The petiole is marginally inserted and flexible, as inferred from the sharp angle observed in specimen STRI 12472 (Figure 5B). A single vascular strand in the petiole is interpreted from the same specimen, observed as a single carbonized thickening along the petiole (Figure 5B). Laminas vary from medially asymmetric (Figure 5A) to symmetric (Figure 5F), and their base insertion varies from asymmetric (Figure 5B) to nearly symmetric (Figure 5D,E).

The leaf venation is pinnate with subopposite, brochidodromous secondary veins, distinctively crowded at the base. Their attachment angle to the midvein is consistently acute. Agrophic veins are compound and arise from the two basalmost pairs of secondary veins. Intersecondary veins are absent. Tertiaries are noticeably chevroned percurrent proximally, becoming straight percurrent distally (Figure 5D). Epimedial tertiaries are opposite percurrent with a proximal course perpendicular to the midvein and distally basiflexed, and external tertiaries are looped. Fourth‐ and fifth‐order veins are reticulate. Areolation is moderate to well developed, and freely ending veinlets are simple.

***Etymology***—The specific epithet refers to “Bacatá,” the original name of the main settlement of the Muisca people in the Eastern Cordillera's Altiplano in pre‐Columbian times.

***Holotype***—STRI 45845 (Figure 5).

***Type locality and age***—Locality MC1813 (4.534366°, −74.139274°), Ceragrés mine, Bogotá Formation, Paleocene (Selandian–Thanetian), Bogotá, Cundinamarca, Colombia.

***Repository***—Universidad de Caldas, Manizales, Colombia.

***Additional specimens examined***—Universidad Eafit, Medellín: STRI 12462, STRI 12463, STRI 12465, STRI 12466, STRI 12468, STRI 12469, STRI 12470, STRI 12471, STRI 12472, STRI 12473, STRI 12673, STRI 12674, STRI 13023; Universidad de Caldas, Manizales: STRI 47091, STRI 47096, STRI 47097, STRI 47098, STRI 47099, STRI 47101, STRI 47102.

***Systematic affinity***—*Goweria* was described from the Puget Group in Washington (Wolfe, 1968) as having ovate to lanceolate leaves with asymmetric bases, five basal veins (originally interpreted as primary veins using common leaf terminology of the time), straight secondary veins that loop close to the margin, widely spaced tertiaries, a marginal secondary vein, and thick petioles. The genus was originally described under Menispermaceae, based on the occurrence of a marginal secondary vein, but was later reassigned to Icacinaceae (Wolfe, 1977), as the former was based on a misinterpretation of a thickened leaf margin in the type specimens as a marginal vein. This reassignment was supported by Allen et al. (2015), who described *Goweria bluerimensis* S.E. Allen, Stull, & Manchester on the basis of leaves from the Eocene Bridger Formation in Wyoming and compared these to extant Icacinaceae, primarily *Iodes*, upholding *Goweria* as a form‐genus of leaves of the family. Recent revisions of the fossil record of Icacinaceae have considered the affinity of most *Goweria* species to Icacinaceae as dubious but highlight *G. bluerimensis* as the most convincing assignment to the family (Del Rio et al., 2020).

We follow Wolfe (1977) and Allen et al. (2015) in describing *Goweria bacatana* under Icacinaceae. Allen et al. (2015) noted that the flexible petioles of Icacinaceae bear a single vascular strand, as observed in *G. bluerimensis* and *G. bacatana*, and suggested this to be indicative of Icacinaceae. Aside from the occurrence of a single petiolar vascular strand, we highlight that the variation in base shape and symmetry of *G. bacatana* supports an affinity to the family. It is not uncommon to find leaves that vary from symmetric to slightly asymmetric within the same individual in species in the genera *Casimirella* Hassl, *Iodes*, and *Notapodytes* Blume, among others. While variation in leaf symmetry and shape may be related to shoot plagiotropic versus orthotropic growth (e.g., Yakang et al., 2023), Icacinaceae is an architecturally rich family (Hallé, 1971) and these patterns are therefore not expected to be consistent across it.

**Comparison to other fossil** ***Goweria***—Five species in the genus *Goweria* have been described previously, known from Paleogene deposits in North America and Japan (Table 1). *Goweria bacatana* can be distinguished from all these, requiring the need for a new species. *Goweria dilleri* (Knowlton) Wolfe and *G. linearis* Wolfe, described from the Puget Group in Washington, differ from *G. bacatana* in having abundant intersecondary veins (not present in *G. bacatana*) and in having greater length:width ratios. *Goweria linearis* also differs in apex shape and in having irregular teeth (originally described as bumps along the leaf margin). *Goweria alaskana* Wolfe from the Eocene Kushtaka Formation in Alaska and *G. bibaiensis* Tanai from the middle Eocene of Hokkaido both differ in having camptodromous veins and marginal tertiaries that occasionally form teeth (Wolfe, 1977; Tanai, 1990). This contrasts with the strictly looping secondary veins and untoothed margin of *G. bacatana*. The Eocene *G. bluerimensis* differs in having an ovate shape and a rounded, obtuse base, unlike the elliptical shape and acute base of *G. bacatana*.

**Table 1:** Comparative morphology of species in genus *Goweria*.

Taxon	Leaf size	Leaf shape	Length:width	Apex shape	Base shape	Base symmetry	Primary venation	Number of basal veins	Secondary venation	Intersecondary veins	Tertiary veins	Marginal secondary vein	Teeth	Reference
*Goweria bacatana* sp. nov.	Notophylls‐Mesophylls	Elliptical	2:1	Acute‐acuminate	Acute‐cordate	Symmetrical/Asymmetrical	Pinnate	3–5	Brochidodromous	Absent	Percurrent	Absent	Untoothed	This study
*Goweria bluerimensis* S.E.Allen, Stull, & Manchester	Nanophylls‐Mesophylls	Ovate‐elliptical	3:2	Acute‐straight	Obtuse‐cordate	Symmetrical/Asymmetrical	Pinnate	≥5	Brochidodromous	Absent	Percurrent	Present	Untoothed	Allen et al. (2015)
*Goweria dilleri* (Knowlton) Wolfe	Notophylls	Elliptical	3:1	Acute‐straight	Acute‐?	Symmetrical	Actinodromous	3	Brochidodromous	Frequent	Percurrent	Absent	Irregular teeth present	Wolfe (1968); Wolfe (1977)
*Goweria alaskana* Wolfe	Notophylls	Ovate	3:2	Acute‐?	Obtuse‐cordate	Symmetrical	Pinnate	5	Brochidodromous	Frequent	Percurrent	Absent	Irregular teeth present	Wolfe (1977)
*Goweria linearis* Wolfe	Notophylls	Ovate‐falcate	4:1‐5:1	Acute‐acuminate	Obtuse‐rounded	Symmetrical/Asymmetrical	Pinnate	3	Brochidodromous	Frequent	Percurrent	Absent	Irregular teeth present	Wolfe (1968)
*Goweria bibaiensis* Tanai	Notophylls	Ovate	2:1	Acute‐straight	Acute‐rounded	Symmetrical	Actinodromous	5	Brochidodromous	NA	Percurrent	Absent	Irregular teeth present	Tanai (1990)

## DISCUSSION

The morphology and anatomy of *Punctiuga bachue*, *Palaeophytocrene paramoae*, and *Goweria bacatana* support recognition of these fossil species and their placement within the family. Endocarps of *Punctiuga bachue* and *Palaeophytocrene paramoae* are recognized on the basis of size, shape, surface ornamentation, and vascularization. Leaves of *G. bacatana* are distinguished by having a petiole with a single vascular bundle, symmetric to basally asymmetric leaf laminas, and basally crowded secondary veins. Together, these new records show a greater diversity of Icacinaceae than was previously acknowledged in the Paleocene tropics.

### Paleoecology of Paleocene rainforests in northern South America

During the Paleocene, lowland Neotropical rainforests characterized by a closed, multistratal canopy (Graham et al., 2019) dominated by angiosperms (Carvalho et al., 2021b) and a family‐level composition resembling that of modern Neotropical forests (Wing et al., 2009) became established in northern South America. Along with the Cerrejón paleoflora from northern Colombia, the Bogotá paleoflora is one of two Paleocene (Selandian–Thanetian) compression floras from northern South America that depict early stages in the assembly and evolution of modern‐like Neotropical rainforests. Both assemblages share the same patterns of family‐level dominance of trees seen in lowland Neotropical forests today (Burnham and Johnson, 2004; Carvalho et al., 2021b) and are characterized by potential high‐canopy elements such as Fabaceae (Herrera et al., 2019), Lauraceae, Annonaceae, Euphorbiaceae, Malvaceae (Carvalho et al., 2011; Puente‐Santos et al., 2026), Melastomataceae (Carvalho et al., 2021a), and Ulmaceae (Herrera et al., 2014); monocots, likely of low‐canopy and epiphytic habits, including Arecaceae (Gomez‐Navarro et al., 2009) and aroids (Herrera et al., 2012); and multiple families with mostly climbing habits such as Menispermaceae (Doria et al., 2008; Herrera et al., 2011), Vitaceae (Herrera et al., 2024), and Icacinaceae (Stull et al., 2012). The presence of the last group has previously been recognized on the basis of endocarps of *Palaeophytocrene* (*P. hammenni*) in the Bogotá Flora and cf. *Phytocrene* sp. at Cerrejón (Stull et al., 2012). The new endocarps and leaves described from the Bogotá Formation confirm the occurrence of Icacinaceae in the Neotropics during the Paleocene and show a previously unknown diversity at that time. Most species in Icacinaceae are vines and climbing plants, and these three new records potentially contribute to the diverse assemblage of woody climbers observed in the Bogotá paleoflora.

The occurrence of a diverse assemblage of climbers in the Bogotá paleoflora has been interpreted as evidence of a closed, multistratal canopy in these forests (Herrera et al., 2011). A diverse assemblage of zoochorous and anemochorous disseminules known from this flora offers further support to this interpretation. Even though anemochory is not the primary dispersal syndrome observed in the Bogotá Flora, the occurrence of winged fruits and seeds such as *Hickeycarpum peltatum* Herrera & Manchester (Herrera et al., 2014) resembles a pattern of dispersal mechanisms of extant emergent and canopy dominant trees in modern tropical rainforests (i.e., *Pterocarpum* spp.; Klitgård and Lavin, 2005; Schley et al., 2022).

In modern rainforests, abundant large, fleshy fruits are a critical part of the ecosystem supporting an immense diversity of vertebrates (Fleming et al., 1987). While there are limited records of fossil frugivorous mammals from the Paleocene of South America and the Bogotá Fm., the presence and abundance of fruits such as the endocarps described here suggest not only the existence of closed‐canopy forests comparable to those of today, but also the occurrence of intertrophic interactions among clades of vertebrates and plants.

*Punctiuga bachue* and *Palaeophytocrene paramoae* provide potential new evidence of the zoochorous component of the Bogotá flora. Previously described zoochorous disseminules include fruits of Menispermaceae (Herrera et al., 2011) and seeds of Vitaceae (Herrera et al., 2024). The endocarps of *Punctiuga bachue* and *Palaeophytocrene paramoae* are remarkable for their relatively large size compared to both extant members of the family and other fossil representatives. Today, most Icacinaceae have generally small (~1 cm) endocarps; within *Iodes*, only *Iodes balansae* Gagnep has fruits of comparable size (Sleumer, 1969). *Iodicarpa* was described to include large endocarps of Iodeae from the Eocene Clarno deposits from Oregon, indicating that other large fruits of Icacinaceae have been widely distributed in the past. The divergence in seed size is often related to changes in growth form across angiosperms (Moles et al., 2005), in which trees often have larger seeds than herbs and shrubs. Whether differences in endocarp size (and consequently seed size, given their unilocular nature) may correlate with growth form within Icacinaceae remains to be tested.

The occurrence of these large endocarps in the Bogotá Flora is consistent with broader evolutionary trends in fleshy diaspore size and abundance observed across the Cretaceous and Paleogene (Tiffney 1984; Wing and Boucher, 1998; Eriksson (2008, 2016); Naware and Benson, 2024) as well as in tropical latitudes today (Chen et al., 2016). While Early Cretaceous angiosperms were characterized by small seeds and fruits and herbaceous habits (Tiffney, 1984; Wing and Tiffney, 1987; Eriksson et al., 2000), diaspores in Late Cretaceous floras vary in size, likely reflecting a higher diversity of growth forms, life‐history traits, and increasing trends in photosynthetic capacity during this time (Naware and Benson, 2024). In a recent review, Naware and Benson (2024) found that the size distribution of fleshy fruits observed in low‐latitude Campanian floras is comparable to those of Paleocene or Eocene age, indicating that large fruit sizes were already common before the end‐Cretaceous in tropical sites. Comparable size distributions in mid‐ and high‐latitude floras are instead observed only by the Paleocene. Surprisingly, compared to low‐latitude floras, fleshy fruits were most abundant in middle to high latitudes during the Paleocene and early to mid‐Eocene, potentially highlighting latitudinal differences in community assembly and plant‐frugivore coevolutionary dynamics. However, this pattern may reflect historical sampling biases, rather than a true evolutionary pattern. *Punctiuga bachue* and *Palaeophytocrene paramoae* provide two new records of fleshy fruits from the Paleocene tropics, increasing the proportion of fleshy fruits in low‐latitude Paleocene floras from 0.38 (10 fleshy fruits out of 26 known fruits) to 0.43, a value closer to that observed in mid‐ and high‐latitude floras (0.52; see Naware and Benson, 2024). This large proportional increase is caused primarily by the relatively low number of known fossil fruits from low latitudes. Further paleontological research in low‐latitude Paleocene floras is needed to confirm this trend.

### Evolution and paleobiogeographic implications

At least five different endocarps in Icacinaceae are now recognized from the Paleocene of northwestern South America, including members of Phytocreneae and Phytocreneae+Iodes. This diversity of Icacinaceae in the Neotropics parallels broad patterns of diversification observed in north mid‐latitudes during the Paleocene (Pigg et al., 2008; Stull et al., 2012; Del Rio and De Franceschi, 2020a). The earliest records for Icacinaceae date back to the Campanian of California (Atkinson, 2022) and the Maastrichtian of Germany (Knobloch and Mai, 1986; Mai 1987). Seventeen species of Paleocene fruits and flowers in Icacinaceae are currently recognized (Figure 1A) from Europe, North America, North Africa, tropical South America, and Patagonia, highlighting their widespread occurrence though low abundance (Crane et al., 1990; Stull et al., 2012) during that time. During the Eocene, the local abundance and number of species in Icacinaceae increased, including representatives of multiple modern genera (Del Rio and De Franceschi, 2020a). This pattern has been interpreted as favored by the expansion of megathermal (Boreotropical) forests into mid‐latitudes during the Eocene (Pigg et al., 2008; Stull et al., 2012; Del Rio, 2018), when global temperatures reached their maximum in the Cenozoic (Judd et al., 2024). An increasing number of Paleocene Icacinaceae in tropical South America is consistent with an early Paleogene radiation and matches inferences based on angiosperm phylogenetic reconstructions that highlight crown‐group diversification of tropical lineages following the end‐Cretaceous extinction event (Ramírez‐Barahona et al., 2020).

Phytocreneae and Iodeae are today restricted to the Old World tropics (Gan et al., 2008; Stull et al., 2015). Nonetheless, their distinctive endocarp morphology has allowed the identification of numerous fossil occurrences documenting a broader distribution in the past (Del Rio and De Franceschi, 2020a). *Punctiuga bachue* and *Palaeophytocrene paramoae* in the Paleocene Bogotá Formation indicate the occurrence of an extinct, potential stem lineage to these clades and confirm the presence of Phytocreneae in northern South America during the Paleocene. In particular, *Palaeophytocrene paramoae* further documents the past widespread distribution of a group that is today restricted to tropical Africa, Madagascar, and Indo‐Malesia (Sleumer, 1971). Recent discoveries of *Palaeophytocrene* from early Paleocene sediments of Patagonia (Poore et al., 2023) and middle‐late Eocene deposits in Australia (Rozefelds et al., 2021) indicate possible dispersal routes and biogeographic connectivity for Phytocrenae via high southern latitudes. Nonetheless, evidence for biogeographic connections between tropical and southern South America during the Paleocene remains sparse. Recent taxonomic revisions of the Danian Salamanca and early Eocene Laguna del Hunco paleofloras show strong biogeographic connections between Patagonia and modern Australasian floras during the Paleogene (Wilf et al., 2005; Gandolfo et al., 2011; Wilf, 2012; Carvalho et al., 2013; Jud et al., 2018; Pujana et al., 2020; Jud and Gandolfo, 2021; Matel et al., 2022). Fruits of *Physalis* (Wilf et al., 2017) and Malvoideae (Siegert et al., 2024) from the early Eocene Laguna del Hunco may represent a connection to tropical floras.

Previous interpretations based on shared elements between Paleocene floras from tropical South America and North America have suggested floristic exchange between both continents during the Paleogene (Jaramillo and Dilcher 2001; Herrera et al., 2011; Stull et al., 2012), though the timing, directionality, and exact route remain unclear. Biotic connectivity between South America and North America could have been favored through the brief establishment of a Caribbean volcanic arc during the Paleocene (Bayona et al., 2011; Cardona et al., 2011), with the emergence of the Central American arch during the middle‐late Eocene (Montes et al., 2012), or through the proto–Greater Antilles during the late Eocene (Iturralde‐Vinent and MacPhee, 1999). Determining whether the occurrence of Phytocreneae in northern South America is reflective of biogeographic connections through the north, the south, or both will require a thorough sampling and detailed taxonomic assessment of Paleogene South American floras.

## CONCLUSIONS

This study describes three new species in Icacinaceae from the Paleocene Bogotá Fm in Colombia, supporting an early Cenozoic diversification for the family in low latitudes. The record of Icacinaceae in the Paleocene floras of northwestern South America reinforces the idea that these Neotropical rainforests supported a diverse assemblage of woody climbers and potentially zoochorous components early in their history. These findings highlight the importance of Paleocene floras in understanding the evolutionary history of Icacinaceae and the development of Neotropical forests. Additional fossil discoveries from underexplored and understudied regions in South America and Central America will refine our understanding of the biogeographic and ecological dynamics that shaped the distribution of this family.

## AUTHOR CONTRIBUTIONS

M.R.C. and F.H. conceptualized the project and collected the fossil material. L.M.P.S., E.A.S., and M.R.C. collected and analyzed the data. L.M.P.S. and M.R.C. led the writing with contributions from all authors.

## Data Availability

All CT scan data produced in this study are available through the University of Michigan Deep Blue repository: 10.7302/c7hv‐tf61 (UR‐CP‐0634); 10.7302/wqgv‐df93 (UR‐CP‐0626); 10.7302/v9ks‐sj52 (UR‐CP‐0632); 10.7302/n2en‐pj32 (MICH‐1461144); 10.7302/yw3q‐zn58 (MICH‐1461090); 10.7302/ksmv‐9r91 (MICH‐1506119); 10.7302/d5y2‐bs57 (MICH‐1506112).
